# The Effectiveness of Acupuncture Compared to Loratadine in Patients Allergic to House Dust Mites

**DOI:** 10.1155/2014/654632

**Published:** 2014-06-05

**Authors:** Bettina Hauswald, Christina Dill, Jürgen Boxberger, Eberhard Kuhlisch, Thomas Zahnert, Yury M. Yarin

**Affiliations:** ^1^Clinic of Otorhinolaryngology, Department of Medicine, University Hospital Dresden, Fetscherstraße 74, 01307 Dresden, Germany; ^2^Institute for Medical Informatics and Biometry, Department of Medicine, University Hospital Dresden, Fetscherstraße 74, 01307 Dresden, Germany

## Abstract

*Background*. The aim of this work was to evaluate the clinical effectiveness of acupuncture and its impact on the immune system in comparison to loratadine in the treatment of persistent allergic rhinitis caused by house dust mites. *Methods*. In this study, 24 patients suffering from persistent allergic rhinitis induced by house dust mites were treated either with acupuncture (*n* = 15) or with loratadine (*n* = 9). The evaluation of the data was based on the subjective and the objective rhinoconjunctivitis symptom scores, specific and total IgE, and interleukins (IL-4, IL-10, and IFN-**γ**) as markers for the activity of Th1 or Th2 cells. *Results*. The treatments with acupuncture as well as with loratadine were considered effective in the patients' subjective assessment, whereby the effect of the acupuncture tended to be assessed as more persistent after the end of treatment. A change in the specific or the total IgE was not detectable in either group. The interleukin profile showed the tendency of an increasing IL-10 value in the acupuncture group. The results of the study show that the effectiveness of acupuncture is comparable to that of loratadine. *Conclusion*. Acupuncture is a clinically effective form of therapy in the treatment of patients suffering from persistent allergic rhinitis. The results indicate the probability of an immunomodulatory effect.

## 1. Introduction

With a prevalence of 20 to 30%, allergic rhinitis is one of the most frequent atopic diseases in Western Europe [1–3]. It leads to a decrease of the patients' quality of life [4] and causes great cost for medication and social benefits [5].

House dust mites, namely,* Dermatophagoides pteronyssinus* and* D. farinae*, are two of the most common persistent allergens. In 30% of all house dust mite allergies, a development of allergic bronchial asthma with coexisting nasal symptoms is expected. Especially in patients suffering from untreated allergic rhinitis, an exacerbation usually follows 5–15 years after the first occurrence of nasal symptoms [6].

Traditional Chinese medicine (TCM), of which acupuncture is a part, gains in importance as an addition to conventional therapies. According to a report of the World Health Organization of 2002 [7] and to clinical studies [8], acupuncture is ranked among the sufficient methods for the treatment of allergic rhinitis and further allergic diseases such as bronchial asthma [9, 10]. Despite the conventional forms of therapy, 64% of patients suffering from persistent allergic rhinitis (PER) desire acupuncture as an alternative form of therapy [11]. Two of the latest multicentre and randomised trials found evidence for a significant improvement of symptoms and the quality of life through acupuncture in patients with allergic rhinitis [1, 12].

Acupuncture, which is described as immunomodulatory therapy [13, 14], has already been examined for its effect and tested for cellular [15] and humoral [10] components of the immune system. In the last decade, numerous findings concerning the key role of CD4+ cells were made. Depending on the subtype, very different cytokines are produced. These can be regarded as prospective markers for the effectiveness of the therapy. Th1 cells mainly express the cytokine interleukin-2 (IL-2) and interferon- (IFN-)*γ*, which cause a cellular immune response, while Th2 cells release interleukins (IL) 4, 5, 10, and 13, which control the maturation of B lymphocytes to cells producing antibodies and their total IgE [16–19].

Examinations show that IL-10 can be considered as markers in the course of the therapy [20]. In several studies on bronchial asthma [10, 21, 22] and allergic rhinitis [23–25], it was possible to show in animal testing as well as in patients that the cytokine profile of IL-10 can be modulated through acupuncture. At the same time, an improvement of the symptoms could be observed. Only fragmentary data are available on the effect of acupuncture on interleukins 4 (IL-4) and 5 (IL-5) and INF-*γ* which are involved in the Th1/Th2 equilibrium [10, 24].

It was the objective of this investigation to prove the effectiveness of acupuncture in the treatment of PER by comparing it with the effectiveness of antihistaminic loratadine as well as to gain a better understanding of the mechanisms of action of acupuncture through the examination of the interleukin profile.

## 2. Material and Methods

Patients in the outpatient department for allergy of the ENT clinic in Dresden suffering from PER were included in this study if a house dust mite allergy was ascertained by means of specific symptoms, skin prick test, and the assessment of the allergen specific IgE. With regard to the skin prick test (Allergopharma Joachim Ganzer KG, Reinbek), a sensitisation to* Dermatophagoides pteronyssinus* or* D. farinae* was then given if the diameter of the wheal measured ≥3 mm after 20 minutes [4]. The CAP-FEIA system of Pharmacia & Upjohn Diagnostics AB, Uppsala, Sweden, was used for the allergological in vitro diagnostics for the determination of the total and the specific IgE. A total IgE of more than 100 kU/L as well as a specific IgE of more than 0.70 kU/L (CAP class 2) [5, 26, 27] was used to verify a sensitisation.

The cytokine profile was examined through the interleukins 4 (IL-4) and 10 (IL-10) and IFN-*γ* using the ELISA technique by Quantikine Immunoassay, Firma R&D Systems, Wiesbaden-Nordenstadt, Germany.

Exclusion criteria were pregnancy, continuous immunotherapy, other therapies influencing the immune system like glucocorticoids or chemotherapeutics, or the use of additional antiallergic medication.

### 2.1. Study Design

This study included 30 patients. The data of 24 patients could be collected fully until the end of the study and were evaluated accordingly (acupuncture group: *n* = 15 and loratadine group: *n* = 9). The average age of the patients was 16.5 ± 9.8 years. The mean duration of the disease was 7.8 ± 6.1 years.  Table 1 shows the mean age of both treatment groups.

### 2.2. Therapy Groups

The patients were randomly assigned to the different treatment groups. Patients being treated with acupuncture received twelve acupuncture sessions in total, two sessions a week, using the same acupuncture points for every patient. Sterile, disposable needles made of stainless steel (Seirin, Dreieich/Germany) were used for body (0.3 × 0.3 mm in strength) and for face and ear acupuncture (0.2 × 0.15 mm in strength).

**Figure 1:**
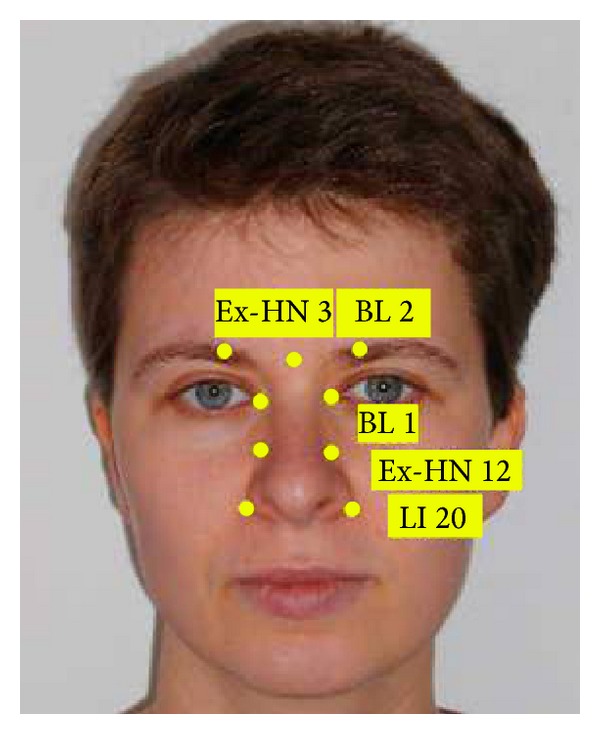
Standardised point chart for facial acupuncture.

**Figure 2:**
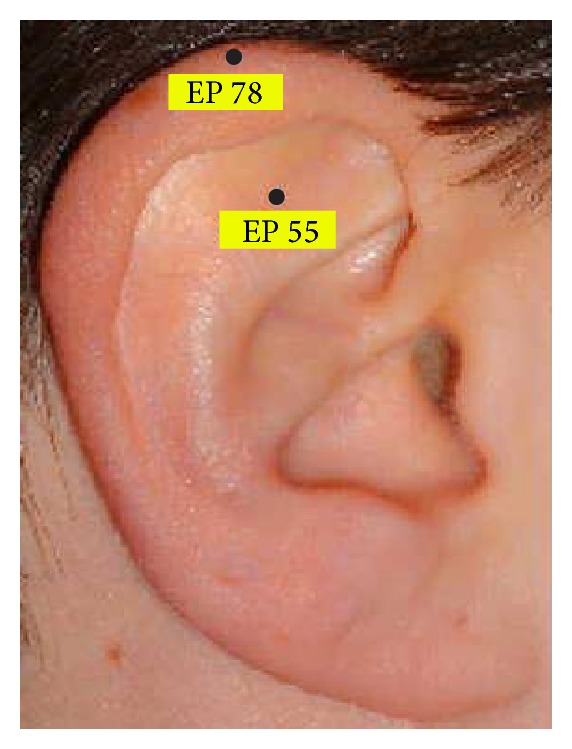
Standardised point chart for ear acupuncture.

**Figure 3:**
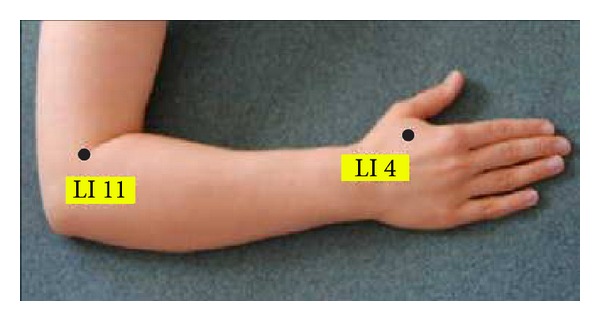
Standardised point chart for “extraordinary” points on the forearm.

To allow for standardisation and comparability, all patients were acupunctured at the same points which were chosen in accordance with the rules of TCM. Needles were inserted unilaterally or bilaterally at the following points: LI 20, Ex-HN 12, Ex-HN 3, BL 1, BL 2, LI 4, LI 11, ear pont 78, and ear point 55 (Figures 1, 2, and 3). In addition, all patients werde acupuntured at LU 20, GB 20, SI 3, and ST 36 (not shown).

The needles were kept in place for 20 minutes. All patients were treated by the same physician throughout the whole study in order to allow for a standardisation.

Patients treated with loratadine took 10 mg of loratadine (Lisino, Essex Pharma GmbH) in the morning of each day over the treatment period of 21 days.

### 2.3. Course of the Study

The patients were examined three times during this study. The first examination took place before the treatment (*t*_1_), the second at the day after the end of treatment (*t*_2_), and the third after an interval of 10 weeks without therapy (*t*_3_).

At all three points, the clinical examination included anterior rhinoscopy, an estimation of the current nasal symptoms by use of the symptom score, and a blood sample (one tube for heparinised whole blood and one for serum each time).

### 2.4. Symptom Scores

All symptom scores were recorded on a 5-point scale (FPS).

#### 2.4.1. Objective Symptom Scores

For the anterior rhinoscopy, the condition of the mucosa and the size of the nasal concha were recorded by the physician. Mucosal reddening and swelling of the inferior nasal concha were rated in the following score: 0 = normal, 1 = slightly changed, 2 = moderately changed, 3 = severely changed, and 4 = most severely changed.

#### 2.4.2. Subjective Symptom Scores

While the objective nasal symptoms and findings were recorded three times in the course of the study, subjective symptoms (complaints) were determined retrospectively in the form of patient interviews. Nasal obstruction and secretion were evaluated using the following scale: 0 = free of symptoms, 1 = slight but noticeable symptoms, not interfering with daily activities, 2 = moderate symptoms, hardly interfering with daily activities and sleep, 3 = severe symptoms, clearly interfering with daily activities and sleep, and 4 = most severe symptoms, substantially interfering with daily activities and sleep.

The assessment of sneezing attacks was classified into 3 categories: 0 = no sneezing attacks, 1 = rare sneezing attacks, 1-2 sneezing attacks per day, and 2 = frequent sneezing attacks with more than 3 attacks per day.

#### 2.4.3. Total Symptom Score

All symptom scores were summed up to a total symptom score in order to elucidate the therapeutic effect.

### 2.5. Subjective Estimation of the Therapeutic Effect

At examinations *t*_2_ and *t*_3_, the subjective state of health was evaluated by comparing the afflictions prior to the therapy with the current ones (1 = improved and 2 = unchanged or worsened).

### 2.6. Statistics

The data collected were evaluated using the statistical software SPSS Version 21 for Microsoft Windows. The results were given in form of mean ± standard deviation or standard error of the mean. The study was planned as repeated measures design and consequently evaluated by means of an analysis of variance. A significance level of *P* < 0.005 was considered statistically significant.

## 3. Results

**Figure 4:**
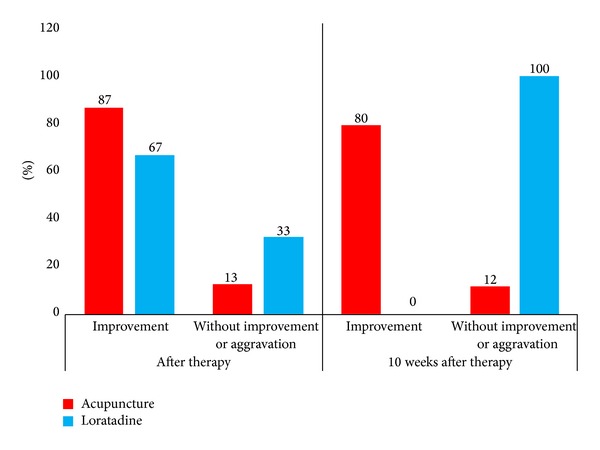
Subjective evaluation of rhinitis symptoms on the day after the end of therapy and after the 10-week therapy-free interval in comparison to the state of health immediately before the beginning of therapy (percentage frequencies in relation to the total patient numbers within the treatment groups).

In the acupuncture group, 87% of the patients reported an improvement of their afflictions at the end of therapy (*t*_1_). 13% did not notice a change at all at *t*_2_ and still not at *t*_3_, 10 weeks after the end of therapy. At *t*_3_, 20% did not notice a change in comparison to the beginning of therapy (*t*_1_) anymore. In the loratadine group, 67% of the patients stated an improvement at *t*_2_, while 33% did not detect an improvement. At *t*_3_ none of the patients treated with loratadine noticed an improvement in comparison to *t*_1_ (see Figure 4).

### 3.1. Total Sum Score

**Figure 5:**
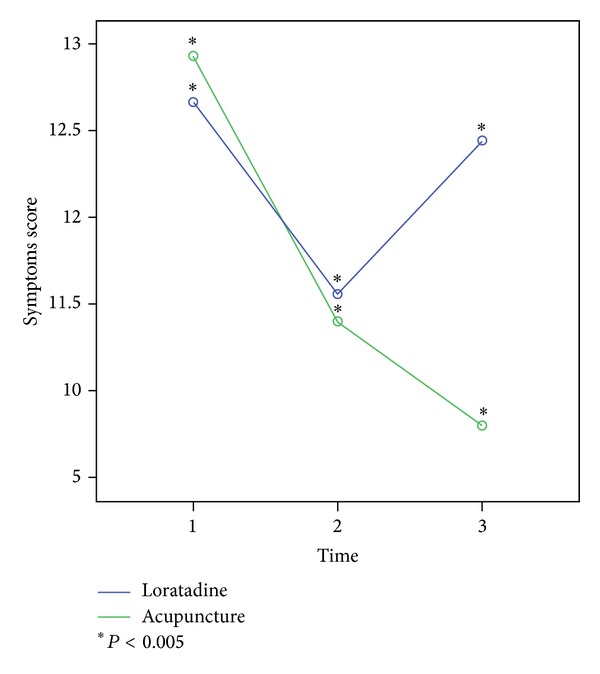
Change in the total symptom scores in both groups during the study period.

Looking at the subjective and objective symptoms separately, there is no significant difference noticeable, neither in the course of the therapy nor between the groups. The total sum score, however, showed significant changes in the time course of the therapy. Both in the acupuncture and the loratadine group, a significant improvement was gained under therapy. In the ten-week period following the therapy, a significant deterioration which led to the recurrence of the allergic symptoms was shown in the loratadine group, while the significant improvement of the symptoms persisted in the acupuncture group (multivariate tests *P* < 0.005). Comparing both groups, no significance was ascertainable (see Figure 5).

### 3.2. Allergic Parameter (Total IgE; Specific IgE* Dermatophagoides pteronyssinus*/*D. farinae*)

Neither the acupuncture nor the loratadine group showed a significant difference in the specific IgE or the total IgE.

### 3.3. Interleukin Profile (IL-4 and IFN-*γ*)

The intermediate IL-4 level in the acupuncture group slightly increased during therapy, between *t*_1_ and *t*_2_, from 0.182 pg/mL to 0.185 pg/mL. It then decreased to 0.177 pg/mL during the period without treatment, between *t*_2_ and *t*_3_. Contrarily, in the loratadine group, the IL-4 level already decreased during therapy from 0.13 pg/mL (*t*_1_) to 0.112 (*t*_2_) and increased then to 0.126 pg/mL s (*t*_3_).

In the acupuncture group, the serum level of IFN-*γ* increased between *t*_1_ and *t*_2_ from 4.799 pg/mL to 5.844 pg/mL and decreased between *t*_2_ and *t*_3_ to 4.399 pg/mL. The intermediate IFN-*γ* level of the loratadine group showed a similar course. After the increase from 5.186 pg/mL to 5.664 pg/mL at the beginning of therapy, the IFN-*γ* serum level decreased to 5.504 pg/mL (*t*_3_).

None of the observed differences of the IL-4 and IFN-*γ* were significant.

### 3.4. Interleukin Profile IL-10

For the IL-10 serum level, an increase from 1.001 pg/mL (*t*_1_) to 1.49 pg/mL (*t*_2_) was observed in the acupuncture group. Also, after the end of therapy, an increase to 1.857 pg/mL (*t*_3_) was shown. In the loratadine group, the IL-10 also increased from 1.84 pg/mL (*t*_1_) to 2.013 pg/mL (*t*_2_) but decreased after the end of treatment to 1.909 pg/mL (*t*_1_).

**Figure 6:**
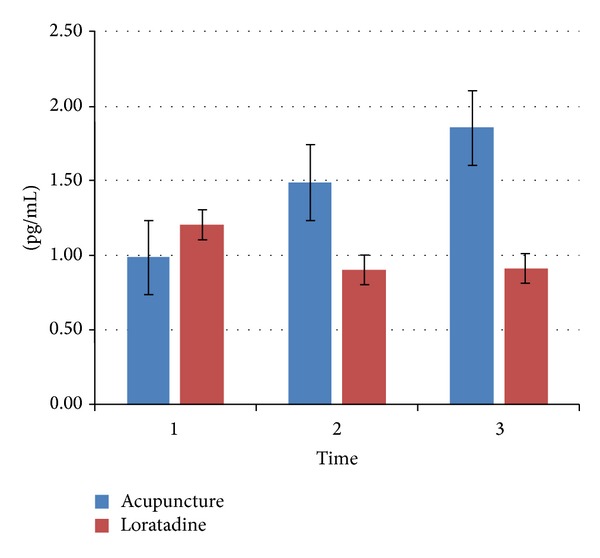
Change in interleukin-10 level in serum; mean values ± standard mean of error at the three examination times.

Even though the increase of the IL-10 serum level was not significant, it showed a distinct tendency to increase in the partial eta squared values (Figure 6).

## 4. Discussion

In order to prove that acupuncture can serve as complementary form of therapy in the treatment of PER, its effectiveness was compared to that of loratadine. It was also examined whether acupuncture has a long-term effect beyond the end of the treatment period. Furthermore, the theory according to which the therapy effect of acupuncture is based on an immunomodulatory effect was checked.

The role of IL-10 in the pathogenesis of allergic diseases during treatment and its level changes in the serum are currently the subject of controversial discussion [20]. It was shown that the IL-10 is able to obstruct the histamine release of activated mast cells [28]. In addition, an increased IL-10 level in the nasal mucosa led to a definite reduction of nasal allergy symptoms of patients with dust mite allergy after nasal provocation [29]. Thus, the IL-10 level could function as a marker for the effectiveness of antiallergic therapy. Some reasearchers suggest that the interleukin levels, especially that of IL-10, change under acupuncture [10, 21, 24].

In our study, we were likewise able to observe that the IL-10 level tends to increase in the acupuncture group. This observation could be an indication for the immunomodulatory effect of acupuncture. Due to the small number of patients, however, it was not possible to show a significance.

Even though there is information that the level of the other cytokines investigated here, namely, IL-4 and IFN-*γ*, can change during acupuncture [10, 21, 24], this was not observed in our study, which might be due to the small sample size.

In a number of studies, the effectiveness of acupuncture in regard to quality of life and the reduction of medication as well as significant improvements of the clinical symptoms has already been shown [1, 8, 12, 30, 31]. Even though our results did not show any significant differences of the single symptoms, a significant improvement of the symptoms was observed in the total sum scores in both the acupuncture and loratadine groups during the course of the treatment. This outcome correlates with the patients' subjective assessment of their state of health recorded immediately at the end of treatment. The difference between both groups develops within the 10-week period without treatment, between *t*_2_ and *t*_3_. While the patients in the acupuncture group still experienced improvement in symptoms (a significant improvement in comparison to the beginning of therapy), the symptoms of the loratadine group started to increase again after the end of treatment. Nevertheless, this difference between both groups was not significant.

Modern medicine requires an evidence-based, double-blind, and placebo-controlled study design in order to prove effectiveness. This is, however, hardly applicable for acupuncture studies. Especially blinding and placebo control present an unsolved problem. Theoretically, a blinding would be possible for laser acupuncture but a comparability of the effectiveness of needle acupuncture with laser acupuncture has not yet been proven. A placebo treatment with acupuncture needles founders on the circumstance that the so-called sham acupuncture, where acupuncture needles are inserted in the acupuncture points, can still have a physiological impact or an effect on the immune system. As the skin is related to internal organs and body systems by the principle of segmental innervations [32], it is not possible to exclude an impact of the skin irritation on the examined effect.

In our study, we tried to prove the effectiveness of acupuncture in PER through changes in the interleukin level in the serum. However, parameters depending on interleukin can be strongly influenced by factors such as autoimmune diseases, inflammations, or even the weather. It might be for this reason that no significant changes in the interleukin level in the serum could be found. Furthermore, a larger number of participants are necessary to prove significance of the treatment effects. The small number of patients allows in many cases only a statistical tendency to increase.

Despite these limitations, the results at hand make it possible to conclude that acupuncture itself and the acupuncture points used are effective in the treatment of PER. Acupuncture, therefore, presents a suitable alternative for patients with drug intolerance or pregnancy. Further studies with a larger patient collective are necessary to confirm these positive results of the mode of action of acupuncture and to examine the effectiveness of further TCM acupoints.

## 5. Conclusion

Acupuncture is an effective, well-tolerated form of therapy in the treatment of patients suffering from dust mite allergy with its effect being comparable to loratadine.

Although the theory that the mechanism of action of acupuncture is based on immunomodulation could not be proven significantly, it was possible to show a tendential increase of the IL-10 level in the serum under acupuncture. For a definite assessment of this issue, further studies with larger numbers of patients are necessary.

Acupuncture can function as an effective therapeutic alternative for patients having a contraindication to specific immunotherapy or to a medicinal symptomatic therapy.

## Figures and Tables

**Table 1:** Demographic structure.

	Acupuncture *n* = 15	Loratadine *n* = 9
Male/female	9/6	3/6
Age (years)	28.1 (±9.9)	24.9 (±9.6)
Duration of disease (years)	7.3 (±6.7)	8.6 (±5.1)

Demographic data: illustration of gender, age, and duration of disease (mean values ± standard deviations) within the groups; *n*: number of patients.

## Abbreviations

EP: Ear point
PER: Persistent allergic rhinitis
TCM: Traditional Chinese medicine
FPS: Five-point scale.
